# Antioxidative Effect of Dihydrosphingosine (d18:0) and α-Tocopherol on Tridocosahexaenoin (DHA-TAG)

**DOI:** 10.1021/acs.jafc.3c02668

**Published:** 2023-09-26

**Authors:** Eija Ahonen, Annelie Damerau, Kaisa M. Linderborg

**Affiliations:** Food Sciences, Department of Life Technologies, University of Turku, Turku 20014, Turun yliopisto, Finland

**Keywords:** carbonyl–amine reactions, antioxidant synergism, tocopherol, omega-3 fatty acid, sphingoid base, dihydrosphingosine, d18:0, imine

## Abstract

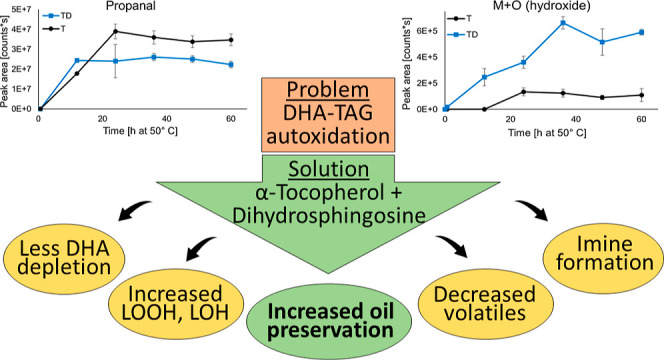

Sphingoid bases have shown promise as effective antioxidants in fish oils together with α-tocopherol, and the effect has been attributed to products resulting from amino–carbonyl reactions (lipation products) between the sphingoid base amine group and carbonyl compounds from lipid oxidation. In this study, the synergistic effect of dihydrosphingosine (d18:0) and α-tocopherol was studied on pure docosahexaenoic acid (DHA) triacylglycerols with an omics-type liquid- and gas-chromatographic mass spectrometric approach to verify the synergistic effect, to get a comprehensive view on the effect of d18:0 on the oxidation pattern, and to identify the lipation products. The results confirmed that d18:0 rapidly reacts further in the presence of lipid oxidation products and α-tocopherol. α-Tocopherol and d18:0 showed an improved antioxidative effect after 12 h of oxidation, indicating the formation of antioxidants through carbonyl–amine reactions. Imines formed from the carbonyls and d18:0 could be tentatively identified.

## 1 Introduction

Docosahexaenoic acid (DHA, 22:6–*n*3) is a polyunsaturated omega-3 (*n*–3) fatty acid with well-documented health benefits. DHA enhances the functioning of the heart, brain, and cardiovascular system, reduces inflammation, improves visual function of infants, and slows down the rate of cognitive decline.^1^ Intake levels of DHA vary considerably between regions, but for most of the world’s adult population, the mean intake is clearly inadequate when compared to recommendations.^2,3^ DHA is mainly obtained from fatty fish and other marine sources or supplements and fortified foods made of them. However, the utilization of DHA is challenging for the food industry due to its high susceptibility to oxidation, which is caused by the high degree of unsaturation of the DHA acyl chain with six double bonds. Oxidation of unsaturated fatty acids causes unpleasant aromas and flavors as well as a decline in the quality and safety of oil. In food oils, oxidation is typically caused by molecular oxygen through free radical chain reaction (autoxidation) with initiation, propagation, and termination steps. In the initiation phase, a free radical abstracts hydrogen from a lipid acyl chain, and an alkyl radical (L^•^) is formed. Molecular oxygen adds to the alkyl radical, forming a peroxyl radical (LOO^•^), which can propagate the reaction by abstracting hydrogen from other lipid acyl chains, forming lipid hydroperoxides (LOOH). Lipid hydroperoxides can decompose by homolytic cleavage to hydroxy- (OH^•^) and alkoxy radicals (LO^•^), and the further cleavage of LO^•^ on either side of the alkoxy carbon leads to the formation of a complex variety of volatile and nonvolatile secondary oxidation products, including aldehydes, alcohols, and alkenes. In the termination step, two radicals combine to form nonradical species.^4^ In addition to the above-described reactions, there are, however, several other competing side reactions taking place simultaneously, so the classical free radical chain reaction alone is nowadays considered to be a too simplified view on lipid oxidation.^5^

One strategy for delaying oxidation is the addition of antioxidants, which can be divided into two groups according to their reaction mechanisms. Primary antioxidants (radical-scavenging antioxidants) are mainly phenolic compounds that can donate hydrogen to free radicals or lipid radicals, thus inhibiting or delaying the initiation or propagation steps. Secondary antioxidants include chelating agents, oxygen scavengers, reducing agents, and synergists. Synergists can increase the antioxidant activity when used as a combination compared to using either compound separately.^6^ Commonly used natural primary antioxidants in omega-3 oils are tocopherols, whose efficacy has been shown to increase by synergistic effects with ascorbic acid, sphingolipids, and phospholipids.^7–11^ The synergism with aminophospholipids has been explained by their ability to regenerate oxidized forms of tocopherols back to their original radical-scavenging state,^12^ alter the physical location of tocopherols, making them more accessible at the site of oxidation,^13^ and/or by the ability to form antioxidative lipation products in the presence of tocopherols, which also can have synergistic effects with α-tocopherol.^14^ Antioxidative lipation products are also known to form in the reactions of amino acids, proteins, and other amine-containing compounds together with carbonyls formed during lipid oxidation. The identities of such reaction products have been examined in previous studies by reacting single carbonyls and amines,^15–21^ with results showing the formation of different substituted and alkyl pyrroles, dihydropyridines, and pyridinium salts. Previous research reporting reaction products from the oxidation of actual oils with a complex set of reactive compounds is scarce. However, pyrrole derivatives have been detected from *n*-octylamine-treated soybean oil, which has been oxidized at 60 °C in the dark,^17^ as well as from phosphatidylethanolamine- and lysine-treated olive oil, which has been oxidized at Rancimat (110 °C).^14,22^

Sphingoid bases are the structural backbone of sphingolipids, which also contain a fatty acid and a polar headgroup. The type and amount of sphingolipids in foods vary considerably, with highest proportions in egg, meat, dairy, and aquatic products. Sphingolipids are structural components of cell membranes where they act also as important signaling molecules.^23^ In three recent studies, sphingoid bases have shown an exceptionally strong synergistic antioxidative effect with α-tocopherol.^10,24,25^ Suzuki-Iwashima et al.^25^ also showed that the reaction products of sphingoid bases dihydrosphingosine (d18:0, Figure 1) and D-*erythro*-sphingosine (d18:1) with pure propanal and acrolein had antioxidative effects. However, the sphingoid base-carbonyl reaction products have not been identified, and their formation has not been shown in oil. There is also no previous research on the effect of d18:0 and α-tocopherol on the complete oxidation pattern of omega-3 oil, which would be necessary to better understand the underlying reaction mechanisms and the total effect in oxidizing oil. For example, it is unclear which of the formed volatile oxidation products is most favorably being consumed in the possible reactions with d18:0, with consequences on the sensory quality of the oil.

**Figure 1:**
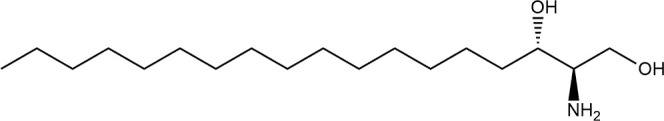
Structure of sphingoid base dihydrosphingosine (d18:0).

This study hypothesized that dihydrosphingosine together with α-tocopherol has a synergistic antioxidative effect on tridocosahexaenoin (DHA-TAG) oxidation due to antioxidative lipation products formed from oxidation product carbonyls and the amine group of d18:0. An omics-type analytical approach was applied to identify the carbonyls taking part in the reaction and the lipation products. One aim was also to examine the effect of dihydrosphingosine and α-tocopherol, combined or one at a time, on the DHA-TAG oxidation pattern in a comprehensive manner. Pure tridocosahexaenoin was chosen instead of natural oil to simplify the set of formed compounds. DHA-TAG was oxidized with α-tocopherol, with d18:0 (selected time points), and with α-tocopherol and d18:0 at 50 °C in the dark. Liquid chromatography (LC) and gas chromatography (GC) methods with mass spectrometric (MS) detection were applied. Volatile and nonvolatile oxidation products were analyzed directly from the oxidized oil as well as after fractionation of the polar phase by solid phase extraction (SPE). α-Tocopherol concentration was analyzed by normal-phase ultrahigh-performance liquid chromatography with fluorescence detection (NP-UHPLC-FLD) and DHA decomposition by gas chromatography coupled with flame ionization detection (GC-FID).

## 2 Materials and Methods

### 2.1 Samples and Oxidation Trial Setup

Tridocosahexaenoin >99% and dihydrosphingosine (d18:0) > 98% were purchased from Larodan (Solna, Sweden), and α-tocopherol >96% was purchased from Sigma-Aldrich (Steinheim, Germany). α-Tocopherol and d18:0 solutions were prepared in ethanol 99.7% v/v (Altia, Rajamäki, Finland). The concentration of the α-tocopherol stock solution was determined according to Podda et al.^26^ with an Evolution 300 BB UV–vis spectrophotometer (Thermo Scientific, Waltham, MA). Tridocosahexaenoin (DHA-TAG) was solvated in *n*-hexane (Honeywell/Riedel de Haën, Seelze, Germany). From the prepared solutions, volumes representing 20 mg of DHA-TAG, 0.01 mg (0.05% w/w) of α-tocopherol, and 0.2 mg (1% w/w) of d18:0 were transferred into 10 mL amber solid-phase microextraction (SPME) vials. The α-tocopherol/d18:0 ratio of 1:20 was chosen due to the enhanced protective effect in volatile analysis by SPME-GC-MS when compared to ratios 1:10 and 1:5 (data not shown). Three sample types (T, TD, and D) were prepared with varying antioxidant compositions. T samples contained α-tocopherol (0.05%), TD samples contained both α-tocopherol (0.05%), and d18:0 (1%) and D samples contained d18:0 (1%). After sample preparation, the vial headspace was gently covered with nitrogen, the vial cooled at −20 °C for 20 min, the cap sealed with parafilm, and the vial moved to −80 °C until the beginning of the oxidation trial. Sample preparation was performed under dim lighting conditions to reduce possible oxidation.

Oxidation trial protocol was adapted from our previous oxidation studies.^27,28^ Right before oxidation, the solvent was evaporated from vials with nitrogen and replaced with compressed air. Oxidation occurred in a Hewlett-Packard 6890 Series Plus G1530A GC oven (Wilmington, DE) at 50 °C in the original SPME vial. T and TD samples were oxidized for 0, 0.6, 12, 24, 36, 48, and 60 h, and D samples were oxidized for 24 and 36 h. The 0.6 h time point corresponded to the SPME time at 50 °C. For each time point, three replicates were prepared for analysis, but later, one replicate for TD12 and one for T24 were excluded from the data as outliers. After the oxidation period, the samples were moved directly to SPME-GC-MS analysis. Right after GC-injection, the sample vial was cooled to room temperature and 2 mL of chloroform/methanol 1:1 (Sigma-Aldrich, Steinheim, Germany: Honeywell/Riedel de Haën, Seelze, Germany) added. Thereafter, 0.8 mL of the sample was filtered with a 0.2 μm PTFE syringe filter (VWR International, Radnor, PA) and divided readily into autosampler vials for the subsequent analysis. The sample part for SPE (1.2 mL) was left unfiltered. Samples were stored at −80 °C until analyzed.

### 2.2 Tocopherol Concentration (NP-UHPLC-FLD)

α-Tocopherol consumption during the oxidation trial was analyzed by the method from Aitta et al.^29^ NP-UHPLC-FLD with a Shimadzu Nexera XR LC-30 HPLC instrument equipped with an LC-20AD XR pump, SIL-20AC autosampler, CTO-20AC prominence column oven, and RF-20A prominence fluorescence detector (Shimadzu, Kyoto, Japan) was used. Excitation and emission wavelengths were 292 and 325 nm, respectively. A Restek Pinnacle DB Silica UHPLC column (1.9 μm, 100 × 2.1 mm, Bellefonte, PA) was applied for an 8 min separation run at 30 °C. Tray cooler temperature was set to 4 °C. The mobile phase (isocratic, 0.4 mL/min) consisted of 2% 1,4-dioxane (Sigma-Aldrich, Steinheim, Germany) and 98% heptane (Honeywell/Riedel de Haën, Seelze, Germany). Samples were transferred to heptane for analysis. For quantification, the α-tocopherol standard curve was prepared from the α-tocopherol stock solution. Limit of detection (LOD) and limit of quantification (LOQ) for the method were 0.08 and 0.26 ng per column, respectively. Chromatographic data were processed by LabSolutions 5.93 (Shimadzu Corporation, Kyoto, Japan).

### 2.3 DHA Concentration (GC-FID)

Fatty acid analysis was done to monitor DHA-TAG depletion during the oxidation trial. Sample oil (0.5 mg) in chloroform/methanol (1:1) and internal standard (0.02 mg) (triheptadecanoin from Larodan, Solna, Sweden) in chloroform (Sigma-Aldrich, Steinheim, Germany) were pipetted into a pyrex vial and evaporated to dryness under nitrogen flow. After solvent evaporation, the fatty acids were transferred into volatile methyl esters by the acetyl chloride/methanol method described by Christie and Han.^30^ Fatty acid content was analyzed by Shimadzu GC-2030 equipped with an AOC-20i autoinjector, a flame ionization detector (Shimadzu Corporation, Kyoto, Japan), and a DB-23 column (60 m × 0.25 mm × 0.25 μm; Agilent Technologies, Santa Clara, CA). Injector and detector temperatures were 270 and 280 °C, respectively. The carrier gas flow (helium) was 2.93 mL/min and the oven temperature program as follows: 130 °C, held for 1 min, raised to 170 °C (6.5 °C/min), raised to 205 °C (2.75 °C/min), held for 18 min, and finally raised to 230 °C (30 °C/min) and held for 2 min. External standards 37 Component FAME mix (Supelco, St. Louis, MO) and 68D (Nu-Check-Prep, Elysian, MN) were used for fatty acid identification, and quantification was done with the internal standard. LOD and LOQ for the method were 0.29 and 0.94 μg/mL, respectively. Data were analyzed by LabSolutions 5.93 (Shimadzu Corporation, Kyoto, Japan).

### 2.4 Nonvolatile Oxidation Products (UHPLC-QTOF)

Nonvolatile oxidation products and the depletion of d18:0 were analyzed by UHPLC-QTOF with a method further developed from Ahonen et al.^27^ Elute UHPLC and Bruker Impact II QTOF instruments from Bruker Daltonic (Bremen, Germany) and a Phenomenex (Torrance, CA) Kinetex 2.6 μm PS C18 column (100 × 2.1 mm) were used. The column oven was set to 30 °C and the autosampler cooler to 10 °C. Samples were diluted 1:1000 (v/v) for the analysis in the original solvent, and 1 μL was injected. A binary solvent system was applied including 95% water (ultrapure from Purelab Chorus instrument, Elga Veolia, High Wycombe, UK) and 5% methanol (Honeywell/Riedel de Haën, Seelze, Germany) as solvent A and 70% 2-propanol (Honeywell/Riedel de Haën, Seelze, Germany) and 30% methanol in addition to 0.1% water as solvent B. Ammonium formate (10 mM, Sigma-Aldrich, Steinheim, Germany) was added to both solvents. In the LC gradient program, the proportion of solvent B was increased from the initial level of 40 to 88% in 5 min, further to 100% in 6 min, held for 2 min, decreased to 40% in 0.5 min, and held for 3.5 min. The total run time was 17 min, and the flow rate was 0.3 mL/min. Electrospray ionization was applied in positive mode. The capillary voltage was set to 4.5 kV, and the end plate offset was set to 500 V. Nebulizer gas pressure, drying gas flow rate, and drying gas temperature were 1.5 bar, 4 L/min, and 350 °C, respectively. Auto MS/MS scanning mode from 60 *m*/*z* to 1200 *m*/*z* was applied. Internal calibration was performed by using sodium formate. For the d18:0 quantification, a standard curve was prepared from a d18:0 stock solution. LOD and LOQ for d18:0 quantification were 0.0007 and 0.002 μg/mL, respectively. MS-DIAL ver.4.92 software,^31^ Bruker Compass DataAnalysis 5.1 (Bruker Daltonic GmbH, Bremen, Germany) and LIPID MAPS database^32^ were used for data processing and identification.

### 2.5 Volatile Oxidation Products (HS-SPME-GC-MS)

Volatile oxidation products were analyzed to track the differences in the oxidative stability and oxidation pattern between the three sample types (TD, T, and D) in real time as the oxidation proceeded. A headspace solid-phase microextraction (HS-SPME) injector with a DVB/CAR/PDMS 50/30 μm (Supelco, Bellefonte, PA) fiber was used. Analytes were separated and detected with a Thermo Scientific GC-MS instrument consisting of Trace 1310 GC, ISQ 7000 mass spectrometer, TriPlus RSH autosampler (Waltham, MA), and a DB-WAX column (60 m × 0.25 mm × 0.25 μm, Agilent Technologies, Santa Clara, CA). The temperature for sample incubation (1 min) and extraction (30 min) was 50 °C. Desorption (5 min) temperature in the GC-injector port was 240 °C (splitless injection) and column oven temperature: 40 °C, held for 2 min, 4.5 °C/min to 110 °C, 2.0 °C/min to 130 °C, 3.0 °C/min to 160 °C, 5.0 °C/min to 225 °C, and held for 2 min. Helium (1.5 mL/min) was used as the carrier gas. Electron ionization (EI) at 240 °C and 70 eV was applied for the MS, and mass-to-charge ratios were scanned between 40 and 300 amu. Compound identification was based on external standards, the NIST MS Search library (version 2.4, National Institute of Standards and Technology, Gaithersburg, MD), and retention indexes. External standards included propanal, 2-ethylfuran, (*E*,*E*)-2,4-hexadienal, and (*E*)-2-pentenal from Fluka (Buchs, Switzerland). Alkane standards C7–C30 (Supelco, Bellefonte, PA) and C5–C12 (Sigma-Aldrich, Steinheim, Germany) were used for calculating retention indexes. Acquired data were processed by the Chromeleon 7.2.9 Chromatography Data System (Thermo Fisher Scientific, Waltham, MA).

### 2.6 Oxidation Products from the Polar Fraction (SPE-GC-MS and SPE-UHPLC-QTOF)

As the concentration of the possibly formed amine-carbonyl reaction products in the samples was expected to be low, SPE with International Sorbent Technology (IST) (Hengoed, Mid Glamorgan, UK) Isolute C18(EC) 100 mg (1 mL) extraction columns was employed for separation and concentration of the polar fraction. The sample (12 mg) in chloroform/methanol (1:1) was evaporated under nitrogen flow and transferred to 300 μL of water/methanol (5:95). The SPE column was solvated with 1.5 mL of methanol and equilibrated with 1.5 mL of water/methanol (5:95) before sample loading. The analytes were eluted to altogether 1.5 mL of water/methanol (5:95), after which the solvent was evaporated at 30 °C under a gentle nitrogen flow. After evaporation, 100 μL of water/methanol (5:95) was added.

For the GC-MS analysis, a Thermo Scientific (Waltham, MA) Trace 1310 GC, AI 1310 autosampler and TSQ 8000 Evo mass spectrometer with a DB-WAX UI column (30 m × 0.25 mm × 0.25 μm, Agilent, Santa Clara, CA) was used. The initial temperature of the column oven was 40 °C, where it was held for 4 min, increased to 240 °C (4.0 °C/min), and held for 7 min. Splitless injection (0.5 μL) at 250 °C was employed with a carrier gas flow (helium) of 1 mL/min. EI at 250 °C and 70 eV was applied for detection with a scanning range from 40 to 350 amu. For the UHPLC-QTOF analysis, 1 μL of the extracted sample was injected. The applied LC and MS methods were as described above (see Section 2.4), except for the LC gradient, where the fraction of B was increased from the initial level of 42 to 100% in 15.5 min, held for 1.5 min, decreased to 42% in 0.5 min, and held for 3.5 min (total run time 21 min). Only the sample time points 0, 12, and 24 h were analyzed for T and TD samples and 24 h for D samples, with three replicates for each. Due to the absence of an internal standard, the results for the SPE extracted samples are shown for each compound as detected/not detected.

### 2.7 Statistical Analysis

Differences in compound levels at each time point were analyzed statistically using the IBM SPSS 28.0.0 statistical software (IBM Corporation, Armonk, NY) by an independent samples *t*-test. *P*-values of *p* ≤ 0.05 were considered statistically significant. A principal component analysis (PCA) plot was produced from the integrated nonvolatile and volatile oxidation product area data (UV scaled) by SIMCA 16 from Sartorius Stedim Data Analytics AB (Umeå, Sweden).

## 3 Results

### 3.1 α-Tocopherol and d18:0 Concentrations

**Figure 2:**
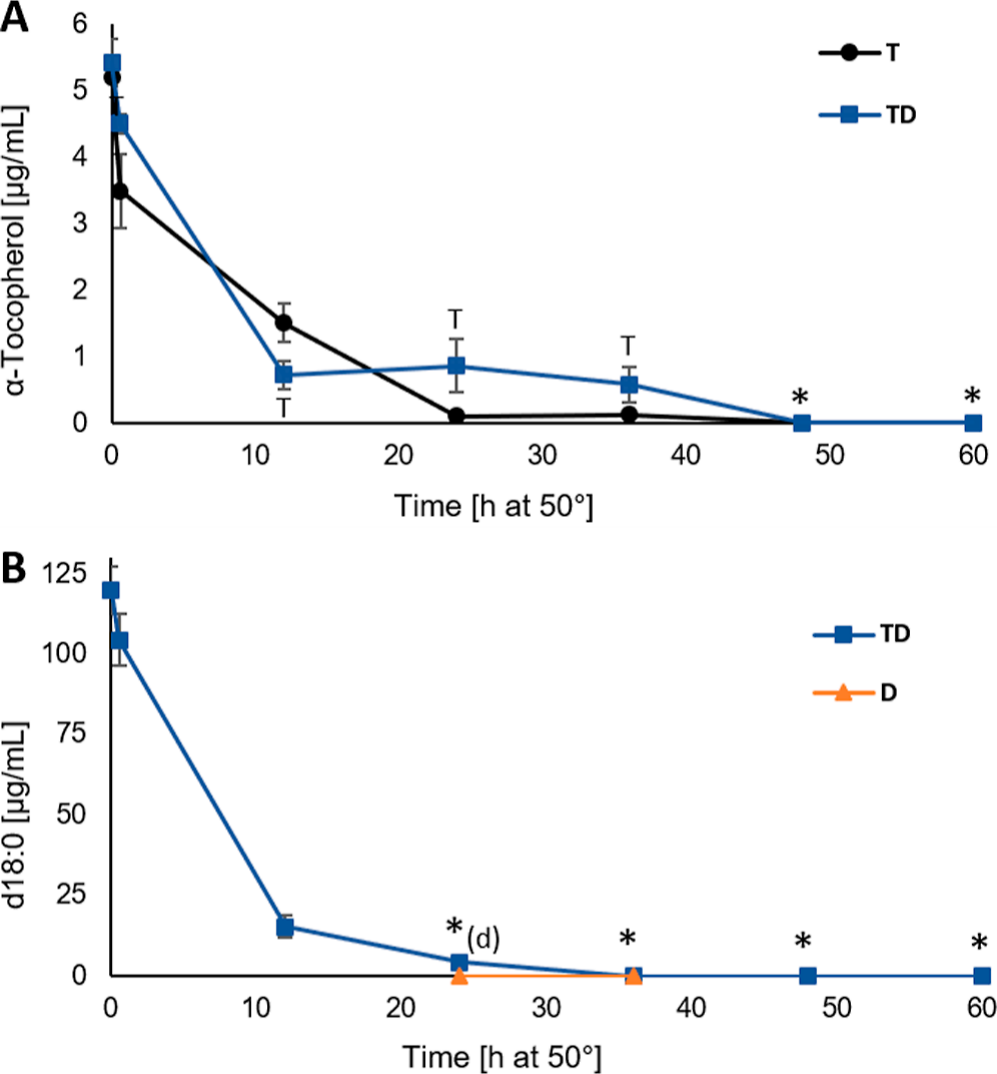
α-Tocopherol (A) and d18:0 (B) concentration (μg/mL) evolution during the 60 h oxidation trial for DHA-TAG samples with α-tocopherol and d18:0 (TD, blue line, square marker), α-tocopherol (T, black line, round marker), and d18:0 (D, orange line, triangle marker). Values are average ± standard deviation (*n* = 3), except for TD12, T24 (*n* = 2), and T0 (*n* = 1). Statistically significant differences between sample types are indicated by the corresponding sample type letter next to the series line. Concentrations < LOD for both sample types are marked with asterisk (*) and for either sample type by asterisk with the corresponding sample type lower case letter *(d/t).

α-Tocopherol concentration evolution during the 60 h oxidation trial is shown in Figure 2A. Concentration decrease was observed from 0 h (totally unoxidized) to 0.6 h oxidized (SPME time). At 12 h, the level was lower in the TD samples (0.72 ± 0.20 μg/mL) when compared to T (1.51 ± 0.29 μg/mL). Thereafter, the level in TD stayed almost constant from 24 to 36 h (0.87 ± 0.41 and 0.58 ± 0.26 μg/mL, respectively), while in the T samples, a decrease to 0.10 ± 0.00 μg/mL at 24 h was observed. At 48 and 60 h, the concentration was below the detection limit for both sample types. Better preservation of α-tocopherol in the presence of d18:0 was also reported by Shimajiri et al.^10^ The quite stable α-tocopherol level from 12 to 36 h in TD might be related to the formation of antioxidative lipation products, which could slow down α-tocopherol consumption and/or regenerate α-tocopherol back to its radical-scavenging state. Depletion of d18:0 in the TD and D samples (selected time points) is shown in Figure 2B. In the TD samples, d18:0 depleted from 116.81 ± 7.14 (unoxidized) to 15.04 ± 3.18 μg/mL in 12 h, and the level further decreased to 4.24 ± 0.11 μg/mL in 24 h. In the D samples, the concentration was below the detection limit at 24 and 36 h. The fast decrease in the d18:0 level occurred simultaneously with the fast α-tocopherol level decrease in the TD samples. The results confirmed that d18:0 rapidly reacts further in the presence of lipid oxidation products and α-tocopherol.

### 3.2 DHA Concentration

**Figure 3:**
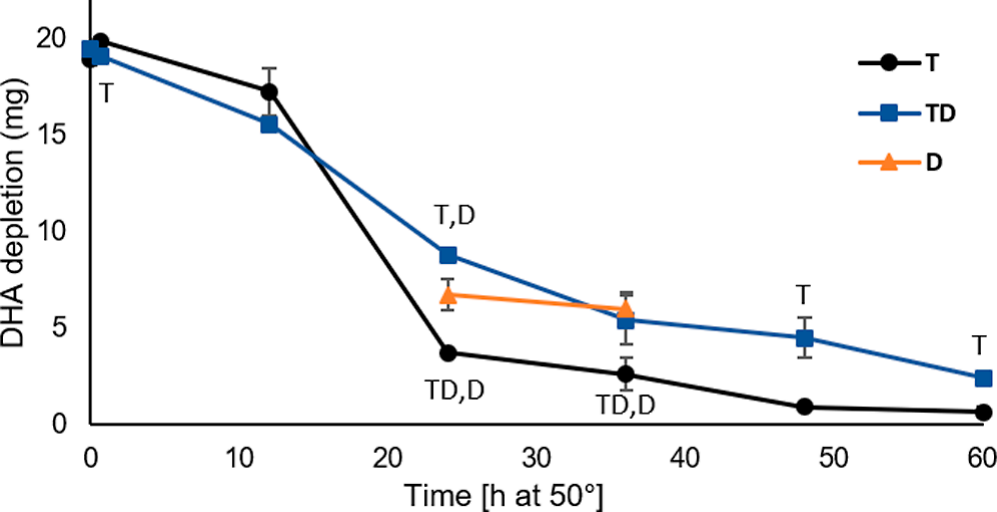
DHA depletion (mg) during the 60 h oxidation trial for DHA-TAG samples with α-tocopherol (T, black line, round marker), α-tocopherol and d18:0 (TD, blue line, square marker), and d18:0 (D, orange line, triangle marker). Values are average ± standard deviation (*n* = 3), except for TD12 and T24 (*n* = 2). Statistically significant differences between sample types are indicated by the corresponding sample type letter next to the series line.

DHA depletion during the oxidation trial is shown in Figure 3. From 0 to 12 h, the oil with both α-tocopherol and d18:0 (TD) was decomposing slightly faster, but after that, it was better preserved when compared to α-tocopherol-containing oil (T). At 24 h, the DHA concentration was 8.82 ± 0.22 mg in TD and 3.73 ± 0.12 mg in T. Better preservation of oil with both d18:0 and α-tocopherol has been reported also earlier.^10^ The concentrations for the two analyzed time points for d18:0 oil (D) were also showing better preservation when compared to T, indicating that d18:0 alone could also have antioxidant potential.

### 3.3 Nonvolatile Oxidation Products

Altogether, 28 oxidation products were tentatively identified in the UHPLC-QTOF analysis (Table 1). For the oxidation products with a cleaved acyl chain, the intensities from the actual analysis were in many cases too low for fragmentation, and thus separate multiple reaction monitoring analysis were performed. For some oxidation products, higher concentrations in the SPE extracted samples enabled identification through fragmentation spectra. For DHA-TAGs, especially the ammonium adduct ([M + NH_4_]^+^), diacylglycerol (DAG) fragments with loss of one fatty acid from the DHA-TAG (loss of RCOOH + NH_3_ from [M + NH_4_]^+^) were explanatory for identification. DAG fragments with one added oxygen (+16 Da) corresponded to *m*/*z* 711.50 (DAG + O) and two added oxygens to *m*/*z* 709.49 (DAG + 2O-H_2_O), *m*/*z* 727.50 (DAG + 2O) and *m*/*z* 693.49 (DAG + 2O-H_2_O-O). The loss of 18 Da and 18 + 16 Da for lipid hydroperoxide ammonium adducts was also noticed by Suomela et al.^33^ when analyzing TAG hydroperoxide reference compounds. The peaks for the masses with the addition of several oxygens to DHA-TAG can contain polyhydroperoxides and cyclic structures as well as additions of several single oxygens. Two separate peaks for the addition of one oxygen (+16 Da) to DHA-TAG could be detected. The compound that eluted first (8.56 min) did not produce any oxidized fragments, while for the second one, the addition of one oxygen (*m*/*z* 711.50) was seen in the DAG fragment. The fragmentation behavior and retention order^33–35^ would suggest that the first compound was hydroxide and the second one an epoxide formed to the site of a double bond. For DHA-TAGs with a cleaved acyl chain, the DHA-DAG fragments could show whether there was oxygen also in the intact DHA chains. As an example, for the TAG 22:6/22:6; O2/6:1; O, no fragments for unoxidized DHA-DAG (*m*/*z* 695.50) were found, but instead the fragments for DHA-DAG + 2O (*m*/*z* 709.49, *m*/*z* 727.50, *m*/*z* 693.49) were detected. In the DAG fragments including a cleaved chain, the masses for 22:6/6:1; O (*m*/*z* 497.32) as well as for 22:6; O2/6:1; O with loss of water (*m*/*z* 511.32, data not shown) were detected. Preliminary carbon number and double bond equivalents for the detected masses were obtained from LIPIDMAPS database with the assumption of DHA being the only fatty acid in the sample TAGs.

**Table 1:** Tentatively Identified Compounds from the UHLPC-QTOF Analysis^a^

compound	mass *m*/*z*	adduct *m*/*z*	adduct	RT min	main fragments *m*/*z*
dihydrosphingosine (d18:0)	301.298	302.306	[M + H]^+^	4.57	284.295_266.284_254.285
d18:0/MDA/d18:0	638.600	639.608	[M + H]^+^	6.52	356.318_338.308_284.297_397.344_266.286
DHA-DAGs (4)					
DAG 22:6/22:6; O4	776.484	794.519	[M + NH_4_]^+^	6.02	385.276_431.246_759.490_741.474
DAG 22:6/22:6; O2	744.494	762.528	[M + NH_4_]^+^	6.41	385.276_711.503_309.224_727.463
DAG 22:6/22:6; O	728.500	746.534	[M + NH_4_]^+^	6.44	385.275_711.502_309.224
DAG 22:6/22.6	712.507	730.540	[M + NH_4_]^+^	7.25	385.272_311.237_293.225
DHA-TAGs (8)					
TAG 22:6/22:6/22:6; O8	1150.696	1168.729	[M + NH_4_]^+^	6.58	385.275_759.488_741.479_725.483_709.488
TAG 22:6/22:6/22:6; O9	1166.706	1184.740	[M + NH_4_]^+^	6.61	385.276_741.478_725.483_759.488_773.467
TAG 22:6/22:6/22:6; O6	1118.709	1136.743	[M + NH_4_]^+^	7.25	385.272_693.491_695.508_709.488_727.498_759.488_309.224
TAG 22:6/22:6/22:6; O4	1086.718	1104.752	[M + NH_4_]^+^	7.79	385.274_695.508_309.224_759.488
TAG 22:6/22:6/22:6; O2	1054.728	1072.762	[M + NH_4_]^+^	8.49	695.503_385.273_709.484_325.225
TAG 22:6/22:6/22:6; O	1038.733	1056.767	[M + NH_4_]^+^	8.56	695.504
TAG 22:6/22:6/22:6; O	1038.733	1056.767	[M + NH_4_]^+^	9.20	695.503_711.502_385.272_309.220
TAG 22:6/22:6/22:6	1022.736	1040.770	[M + NH_4_]^+^	9.92	695.505_385.275_311.238_293.228
DHA-TAGs with Cleaved Acyl Chain (14)					
TAG 22:6/22:6; O2/7:0; O	872.552	890.573	[M + NH_4_]^+^	6.43	385.278_513.325_709.489_309.223_495.271_325.216_693.487
TAG 22:6/22:6; O2/6:1; O	856.552	874.586	[M + NH_4_]^+^	6.46	385.276_693.493_709.495_497.320_309.223_325.220_727.498
TAG 22:6/22:6/8:1; O3	884.584	902.618	[M + NH_4_]^+^	7.02	695.509_385.277_507.314_311.237
TAG 22:6/22:6/11:2; O3	924.615	942.649	[M + NH_4_]^+^	7.15	695.507_547.346_385.272_311.241
TAG 22:6/22:6/4:0; O (oxo)	796.528	814.561	[M + NH_4_]^+^	7.15	469.299_385.277_311.241_695.512_293.230
TAG 22:6/22:6/5:0; O2	828.559	846.592	[M + NH_4_]^+^	7.16	695.507_469.296_385.276_311.239
TAG 22:6/22:6/6:1; O2	840.559	858.592	[M + NH_4_]^+^	7.23	385.277_695.508_513.323_311.239
TAG 22:6/22:6/8:1; O2	868.591	886.625	[M + NH_4_]^+^	7.23	385.277_695.509_541.318_311.239_509.331
TAG 22:6/22:6/14:2; O3	966.625	984.659	[M + NH_4_]^+^	7.26	695.509_385.276_575.342_607.366
TAG 22:6/22:6/6:1; O	824.563	842.596	[M + NH_4_]^+^	7.26	695.502_385.273_479.310_311.237
TAG 22:6/22:6/9:2; O2	880.589	898.625	[M + NH_4_]^+^	7.32	695.503_385.274_553.353
TAG 22:6/22:6/9:2; O (oxo)	862.572	880.606	[M + NH_4_]^+^	7.42	535.346_311.240_225.115_385.276_695.510
TAG 22:6/22:6/12:3; O (oxo)	902.604	920.638	[M + NH_4_]^+^	7.67	575.377_311.241_695.511_385.277_265.147
TAG 22:6/22:6; O2/10:2	894.603	912.637	[M + NH_4_]^+^	7.72	535.344_309.223_385.277_549.340_693.492_709.486

a Compound name, mass (*m/z*), adduct mass (*m/z*), retention time, and main fragments in order of lowering intensity for the designated adduct.

For half of the identified oxidation products with a cleaved acyl chain, the formation could be explained by the reactions following the β-cleavage of alkoxy radicals (LO^•^).^36^ The chain 4:0; O (oxo) could form through the scission of 4-LO^•^ following the arrangement to an aldehyde. 9:2; O (oxo) and 12:3; O (oxo) could form from 10- and 13-LO^•^, respectively, through cleavage on the glycerol side, leading to the formation of an alkyl radical (L^•^), which could further oxidize to hydroperoxide, cleave to LO^•^, and arrange internally to an aldehyde. 6:1; O and 6; 1; O2 could form from the L^•^ (from 7-LO^•^ scission), following the addition of OH or OOH. Similarly 9:2; O2 could form from the L^•^ left after cleavage of 10-LO^•^ by the addition of OOH. For the rest of the detected cleavage products, the formation could not be directly explained by the routes following LO^•^ β-scission. However, they could originate from the cleavage of cyclic structures, which are known to be prevalent in oxidized polyunsaturated fatty acids,^37^ from secondary scissions of the primary β-scission products or from other possible radical reactions.

**Figure 4:**
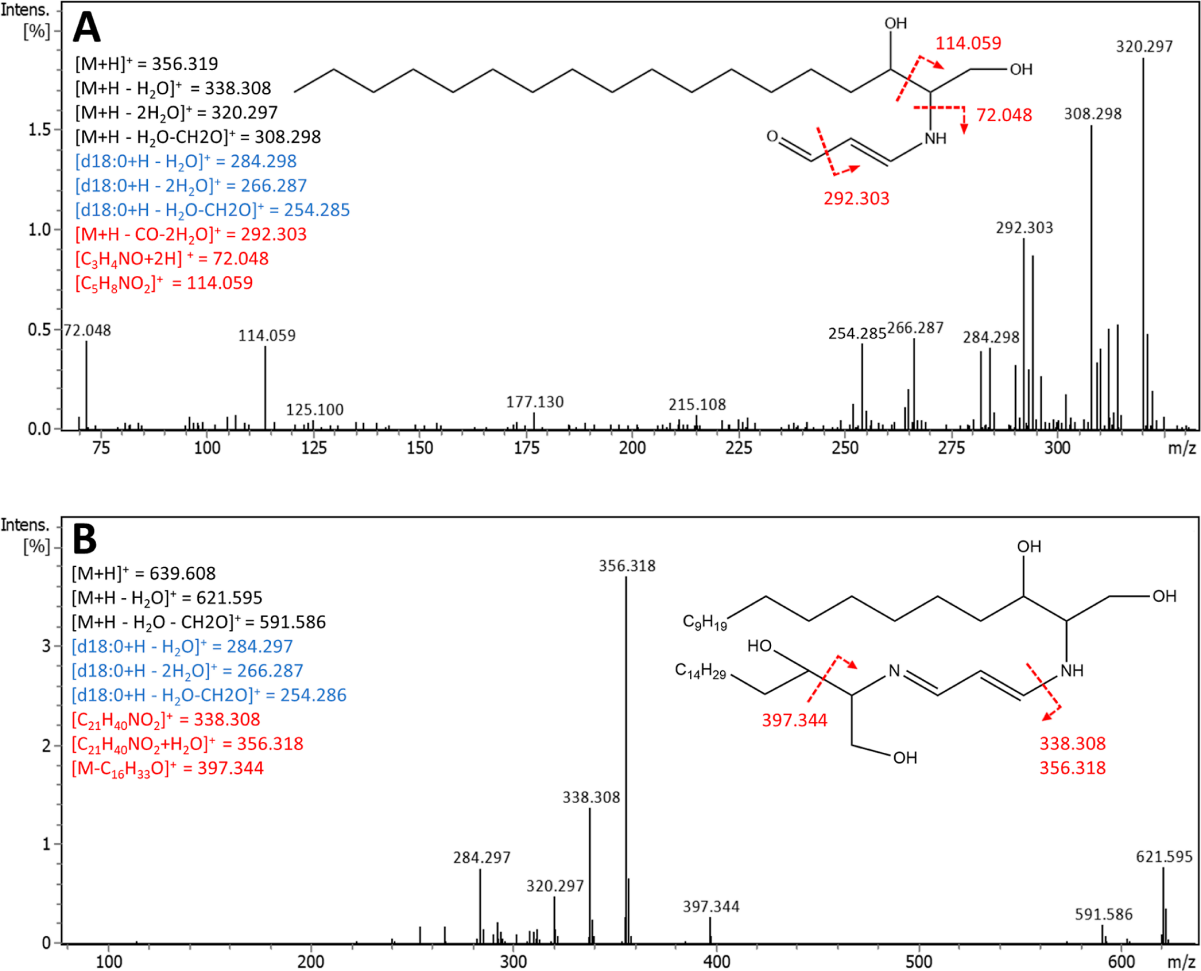
Fragment spectra and suggested fragmentation patterns for tentatively identified MDA imine (d18:0-MDA) (A) and d18:0-MDA-d18:0 (B). Fragments originating from d18:0 are marked in blue, and fragments corresponding to cleavages indicated by arrows in the graph are marked in red. High-intensity molecular ions and for A also [M + H-H_2_O]^+^ are omitted from the spectra.

Identification of the suspected d18:0-malondialdehyde-d18:0 was based on the tentative identification of malondialdehyde (MDA) imine from the SPE-UHPLC-QTOF data of the TD samples. MDA can cross-link with amino compounds like amino acids or peptides.^38,39^ In addition to MDA, also several other possible imine structures could be detected in the SPE data, including masses corresponding to d18:0-imines of, e.g., glyoxal, oxohexadienal, propanal, 2-propenal, and 2,4-decadienal. Since the same mass can correspond to several atomic structures and the α,β-unsaturated carbonyls could also react through Michael addition, the exact identification was not possible in all cases. Possible d18:0-imines and their main fragments are presented in Table S1. Some of these imines seemed to react further rapidly; e.g., the suspected imine for 2-propenal was detected only in the 12 h samples and for propanal only in some of the 0 h sample replicates. Some of the more long-lived imines were detected also in the D 24 h samples in addition to TD, while others were not detected in D despite the abundance in TD at 24 h. The d18:0-MDA-d18:0 was the only nitrogen-containing compound that was detected also in the QTOF analysis of the oxidized samples without SPE. The presumed fragmentation patterns for MDA imine (d18:0-MDA) and d18:0-MDA-d18:0 are presented in Figure 4A,B, respectively. The fragments from d18:0 with losses of water (marked blue in Figure 4) were typical for all of the suspected imine structures detected in the SPE-extracted samples.

**Figure 5:**
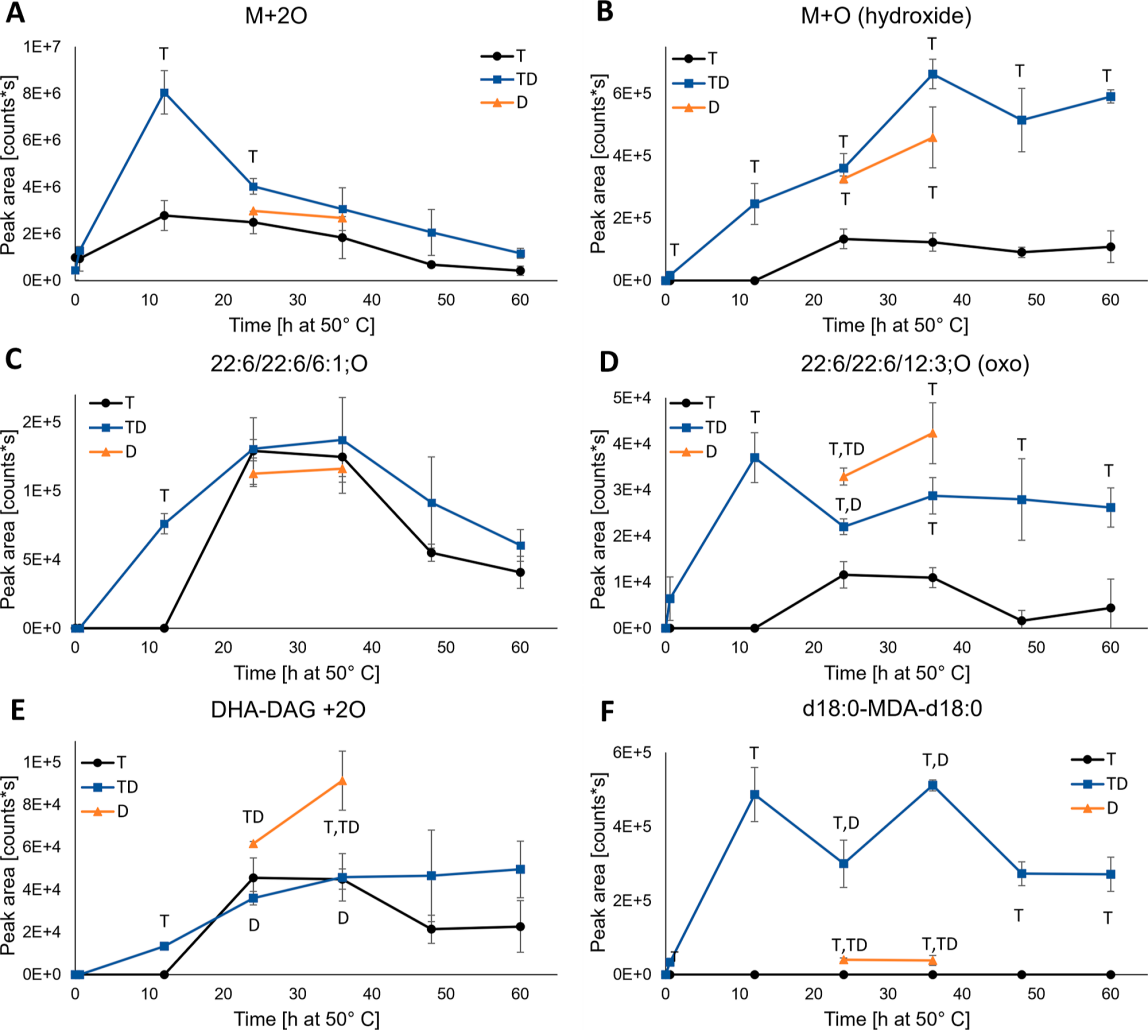
Area evolution of the nonvolatile oxidation products M + 2O (A), M + O (hydroxide) (B), 22:6/22:6/6:1; O (C), 22:6/22:6/12:3; O (oxo) (D), DHA-DAG + 2O (E), and d18:0-MDA-d18:0 (F) for DHA-TAG samples with α-tocopherol and d18:0 (TD, blue line, square marker), α-tocopherol (T, black line, round marker), and d18:0 (D, orange line, triangle marker) during the 60 h oxidation trial at 50 °C in the dark. Values are average ± standard deviation (*n* = 3), except for TD12 and T24 (*n* = 2). Statistically significant differences between sample types are indicated by the corresponding sample type letter next to the series line.

Area evolution for some of the detected nonvolatile oxidation products is presented in Figure 5. Integration was done only for the non-SPE-extracted samples. For the compounds with additions of 2O (Figure 5A), 4O (Figure S1A), and 6O (Figure S1B) to DHA-TAG, the levels were higher in TD samples when compared to T throughout the whole oxidation period, with the most significant difference at 12 h. α-Tocopherol consumption and DHA depletion (Figures 2A and 3, respectively) showed faster oxidation for TD samples from 0 to 12 h, so higher hydroperoxide levels could be expected. However, the substantially higher levels could also indicate higher H donor capacity in the TD samples, which would direct the reactions of LOO^•^ into LOOH instead of other reaction routes, e.g., epoxide formation through LOO^•^ addition to double bonds.^40^ The increased H donor capacity could be due to the antioxidative lipation products. Also, the d18:0 amine moiety is a possible H source for lipid radicals, although the bond dissociation energy exceeds the one from α-tocopherol (92 vs 78 kcal mol^–1^).^40,41^ Similarly, the level of suspected DHA-TAG hydroxide was significantly higher in TD than in T, which could also relate to the higher antioxidant capacity (LO^•^ + H → LOH). For some of the oxidation products with a cleaved acyl chain, the level in T reached the level of TD at around 24 h (Figure 5C), while in the others, it remained lower during the whole oxidation period (Figure 5D), indicating differing reaction routes. In the D samples, the levels of DHA-DAG + 2O (Figure 5E) and 22:6/22:6/12:3; O (oxo) (Figure 5D) were clearly higher than in T or TD at 24 and 36 h; otherwise, the levels were alternating at slightly higher, lower, or in between the levels in T or TD. All the nonvolatile oxidation product levels except for DAG + 2O (Figure 5A–E) were higher in TD when compared to T throughout the oxidation period with basically no induction period, while in the T samples, there was a 12 h induction period for all the oxidation products except for M + 2O. When considering the results of this analysis part alone, it does look like the TD oxidizes faster and produces more nonvolatile oxidation products than T. The observed behavior could, however, also be caused by the rerouting of the oxidation reaction due to higher H availability in TD as stated above. After the increase from 0 to 12 h, the level of 18:0-MDA-18:0 fluctuated at quite a high level (Figure 5F). This oxidation product was not detected in the T samples, and in the D samples, the levels were very low at 24 and 36 h. In the SPE-extracted TD samples, MDA-d18:0 was at its highest level at 12 h, so it could be postulated that the second reaction between the dialdehyde and d18:0 stabilized the compound and inhibited further reactions. The low level of d18:0-MDA-d18:0 in the D samples seemed peculiar, since MDA originates mainly from hydroperoxy epidioxides and bicycloendoperoxides^42^ and was expected to form also in the D samples. The differences in the formed reaction products between D and TD indicate that the presence of α-tocopherol might be needed for the formation or stabilization of some of the lipation products.

### 3.4 Volatile Oxidation Products

**Figure 6:**
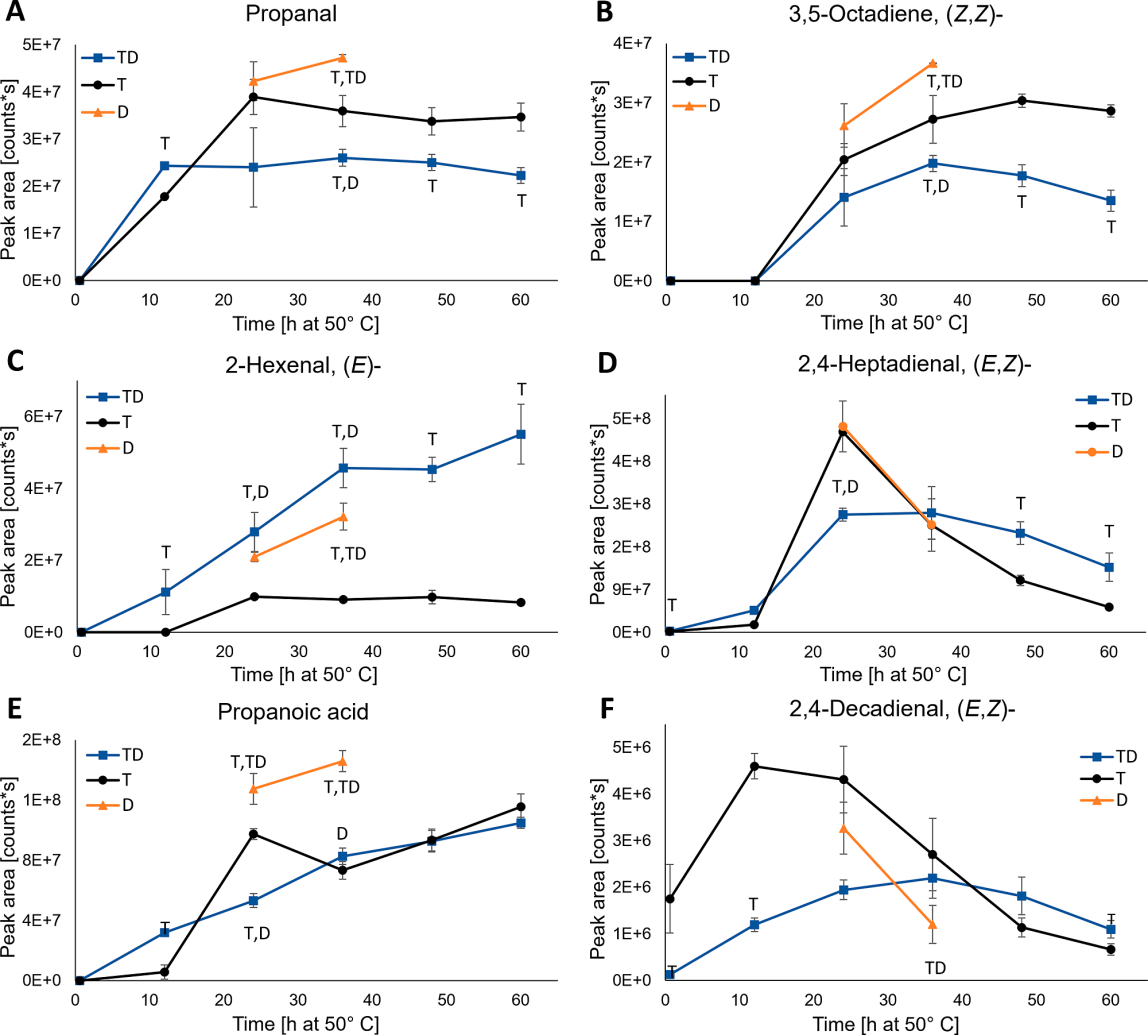
Area evolution of the volatile oxidation products propanal (A), 3,5-octadiene, (*Z*,*Z*)- (B), 2-hexenal, (*E*)- (C), 2,4-heptadienal, (*E*,*Z*)- (D), propanoic acid, (*E*), and 2,4-decadienal, (*E*,*Z*)- (F) for the DHA-TAG samples with α-tocopherol and d18:0 (TD, blue line, square marker), α-tocopherol (T, black line, round marker), and d18:0 (D, orange line, triangle marker) during the 60 h oxidation trial at 50 °C in the dark. Values are average ± standard deviation (*n* = 3), except for TD12 and T24 (*n* = 2). Statistically significant differences between sample types are indicated by the corresponding sample type letter next to the series line.

Altogether 63 volatile oxidation products were detected in the HS-SPME-GC-MS and SPE-GC-MS analyses (Table 2). Twelve of them were detected exclusively after SPE-GC-MS, 19 in both analysis and the majority, 32, only in the SPME-GC-MS analysis. Especially, the early eluting low-molecular-weight aldehydes and alkenes were often missed by the liquid injection GC-MS. These readily volatile compounds are easily concentrated to the SPME fiber. Also, the extraction and evaporation phases of the SPE samples could have led to the loss of some analytes. SPME and liquid injection have been also compared with samples from the same oil, with results showing better sensitivity of SPME for low-molecular-weight analytes and overall higher number of detected compounds.^43^ The biggest compound groups were aldehydes (20 pcs), ketones (14 pcs), and alkenes (8 pcs). Some possibly nitrogen-containing compounds could be detected in both SPME-GC-MS and SPE-GC-MS analyses, but due to small intensities or lack of library match, they could not be identified. Area evolution for some of the identified volatile oxidation products is presented in Figure 6.

**Table 2:** Detected Volatile Oxidation Products from the SPME-GC-MS and SPE-GC-MS Analysis for α-Tocopherol (T) and α-Tocopherol + d18:0 (DT) Containing DHA-TAG Samples (+= Detected in the Sample Type; -= Not Detected in the Sample Type)^e^

compound group	compound	T	TD	match^a^	RT^b^	RI^c^	RI NIST^d^
aldehydes (20)	acetaldehyde	+	+	921	3.8	701	702 ± 12 (82)
	propanal	+	+	911	4.5	790	798 ± 14 (49)
	2-propenal	+	+	981	5.2	845	850 ± 10 (17)
	2-butenal, (*Z*)-	+	+	937	9.3**	1044	1035 ± N/A (1)
	2-butenal, (*E*)-	+	+	924	9.4	1048	1039 ± 7 (26)
	2-pentenal, (*Z*)-	+	+		11.2	1110	
	2-pentenal, (*E*)-	+	+	939	11.9	1134	1127 ± 6 (65)
	3-hexenal	+	+	906	12.3**	1146	1146 ± N/A (1)
	4-heptenal, (*Z*)-	+	+	930	12.6*	1243	1239 ± 9 (78)
	2-hexenal, (*E*)-	+	+	952	14.7	1220	1216 ± 9 (266)
	2,4-hexadienal, (*E*,*E*)-	+	+	916	20.6**	1405	1404 ± 7 (32)
	3,6-nonadienal, (*Z*,Z)-	+	+	906	21.1*	1499	
	2-octenal, (*E*)-	+	+	927	21.5	1431	1429 ± 8 (147)
	2,4-heptadienal, (*E*,Z)-	+	+	912	22.9**	1469	
	2,6-nonadienal, (*E*,Z)-	+	+	882	23.6*	1582	1584 ± 9 (157)
	2,4-heptadienal, (*E*,*E*)-	+	+	928	23.9**	1496	1495 ± 11 (122)
	4-oxohex-2-enal	+	+	928	27.9**	1595	
	2-decenal, (*E*)-	+	-	841	30.0	1647	1644 ± 11 (84)
	2,4-decadienal, (*E*,Z)-	+	+	935	34.9	1769	1755 ± 12 (57)
	2,4-decadienal, (*E*,*E*)-	+	+	945	36.7**	1816	1811 ± 16 (200)
ketones (14)	2,3-pentanedione	+	+	842	6.7*	1057	1058 ± 8 (138)
	1-penten-3-one	+	+	931	8.7**	1023	1019 ± 6 (67)
	3-penten-2-one	+	+	912	11.8	1131	1128 ± 9 (36)
	3-hydroxy-3-methyl-2-butanone	-	+	903	13.0*	1254	1247 ± 6 (6)
	2-propanone, 1-hydroxy-	+	+	851	14.7*	1300	1303 ± 12 (61)
	6-octen-2-one, (*Z*)-	+	+	853	15.7*	1332	1316 ± N/A (1)
	1-hydroxy-2-butanone	+	+	907	19.7	1378	1388 ± 7 (19)
	3,5-octadien-2-one	+	+	930	24.9**	1522	1522 ± 6 (16)
	3,5-octadien-2-one, (*E*,*E*)-	+	+	932	27.0**	1574	1570 ± 7 (37)
	2(5H)-furanone, 5-methyl-	+	+	941	31.6	1684	1664 ± 6 (7)
	dihydro-3-methylene-5-methyl-2-furanone	+	+	890	32.2	1698	
	2,5-furandione, 3,4-dimethyl-	+	+	800	33.5	1734	1714 ± 29 (9)
	2(5H)-furanone, 5-ethyl	+	+	855	34.8**	1767	1748 ± 11 (12)
	2(3H)-furanone, 5-acetyldihydro-	+	+	931	36.1*	2053	2026 ± 13 (3)
alkenes (8)	3-methyl-1,6-heptadiene	+	+	857	5.8	891	
	2,4-octadiene	+	+	916	6.4	920	919 ± 11 (3)
	3,5-octadiene, (*Z*,*Z*)-	+	+	877	6.6	930	
	1,3-(*E*)-5(*Z*)-octatriene	+	+	968	11.0	1102	1108 ± 1 (5)
	1,3,5-(*Z*,*Z*,*Z*)-octatriene	+	+	928	11.0**	1106	1107 ± 15 (2)
	2,4-hexadiene, (*E*,*Z*)-	+	+	941	12.9*	1250	
	5-dodecene, (*Z*)-	+	+	887	15.3	1240	1242 ± 2 (19)
	2-hexene, 3,5,5-trimethyl-	+	+	872	23.6	1487	
acids (6)	acetic acid	+	+	962	22.0**	1446	1449 ± 13 (358)
	formic acid	+	+	927	23.9**	1495	1508 ± 18 (14)
	propanoic acid	+	+	970	25.5**	1538	1535 ± 12 (126)
	butanoic acid	+	+	947	29.4	1632	1624 ± 11 (281)
	3-hexenoic acid, (*Z*)-	+	+	908	41.8	1954	1930 ± 12 (8)
	3-heptenoic acid	+	+	861	43.6		
alcohols (6)	1-penten-3-ol	+	+	844	12.9**	1166	1158 ± 9 (145)
	hex-5-en-3-ol	+	+	833	15.5*	1327	
	2-penten-1-ol, (*E*)-	+	+	911	17.6**	1314	1313 ± 8 (47)
	2-penten-1-ol, (*Z*)-	+	+	952	17.9	1322	1318 ± 7 (67)
	3-hexen-1-ol, (*Z*)-	+	+	906	20.0	1386	1382 ± 9 (358)
	4-decen-1-ol, (*Z*)-	+	-	911	36.2	1802	1791 ± 7 (2)
furans (3)	2-ethylfuran	+	+	910	7.1	954	951 ± 6 (53)
	2-pentylfuran	+	+	818	15.0	1230	1232 ± 9 (166)
	*E*-2-(2-pentenyl)furan	+	+	896	17.2**	1299	1282 ± N/A (1)
benzene derivatives (3)	benzaldehyde	+	+	885	21.6*	1514	1520 ± 14 (459)
	1-propanone, 1-phenyl-	+	+	829	27.4*	1718	1715 ± 19 (7)
	benzene, acetyl	+	+	900	30.4**	1656	1647 ± 13 (127)
esters (2)	formic acid, ethyl ester	-	+	884	4.9	825	824 ± 9 (28)
	octanoic acid, methyl ester	+	+	922	20.0	1388	1385 ± 7 (62)
epoxides (1)	2-ethyl-3-vinyloxirane	+	+	872	8.5	1011	

a NIST match number.

b Retention time (min).

c Kováts retention index.

d NIST retention index, deviation, and number of experimental determinations.

e Some of the oxidation products were detected only in SPE-GC-MS (* after RT), in both analyses (** after RT), or only in SPME-GC-MS (no marking). For the compounds detected in both analyses, the presented identification data is based solely on the SPME-GC-MS analysis data.

With four exceptions, all the identified oxidation products were detected in both sample types (T and TD) (Table 2). For the D samples, data are not shown since only two time points were analyzed. 4-Decen-1-ol (*Z*)- and 2-decenal, (*E*)- were detected exclusively in the T samples. Formic acid ethyl ester and 3-hydroxy-3-methyl-2-butanone were detected exclusively in the TD samples, and the latter one only in the SPE-extracted samples. Area integration was done only for the non-SPE-extracted samples. Evolution of the propanal area (Figure 6A) indicated possible consumption in lipation reactions. There was no induction period in either T or TD samples, and the level increased slightly faster in TD from 0 to 12 h, but after that, it stayed lower in TD throughout the oxidation period. Lower levels at 24 h and thereafter in TD samples were also observed for 2-propenal (Figure S2A), 1-penten-3-one (data not shown), 1-penten-3-ol (Figure S2B), and 1-hydroxy-2-butanone (Figure S2C). Lowest propanal and 2-propenal levels in d18:0- and α-tocopherol-containing samples in comparison to samples containing either compound alone have been also noticed by Uemura et al.^24^ Pure 2-propenal has also been shown to react with d18:0 at 50 °C, producing compounds with an antioxidative effect.^25^ The alkenes 3,5-octadiene (*Z*,*Z*)- (Figure 6B), 3-methyl-1,6-heptadiene (Figure S2D), and 2,4-octadiene (data not shown) had quite a similar level of evolution, where areas for T were constantly higher than for TD, and D was producing even higher levels. For 2-hexenal (*E*)- (Figure 6C), the level was clearly the highest in TD, differing from all the other detected volatiles. This could be due to higher formation in d18:0-containing samples caused by differing reaction routes and/or no consumption in further reactions.

Graph D in Figure 6 for 2,4-heptadienal, (*E*,*Z*)- shows a 12 h induction period for both sample types and higher levels for T and D samples than for TD at 24 h. A similar trend was noticed for 3,5-octadien-2-one (*E*,*E*)- (Figure S2E), 2,4-hexadienal (data not shown), and 4-oxohex-2-enal (data not shown). Participation in carbonyl–amine reactions is also possible for these compounds. For propanoic acid (Figure 6E), a constant increase throughout the oxidation period was seen in both sample types with a 12 h induction period for T samples. For 3-hexenoic acid (*E*)-, constant increase was seen only for the TD samples (Figure S2F). The levels of the ten-carbon aldehydes 2,4-decadienal (*E*,*Z*)- (Figure 6F) and (*E*,*E*)- (data not shown) were significantly higher especially at 12 h in the T samples when compared to TD. The higher consumption of 2,4-decadienal (*E*,*Z*/*E*,*E*)- in TD could be due to carbonyl–amine reactions, which has also been shown before in model systems at higher temperatures.^44,45^ Although the formation of 2,4-decadienal in DHA oil does not directly follow the basic alkoxy radical scission route, it has been detected earlier also in algae- and fish oils rich in omega-3 fatty acids.^46^ The volatile levels in D are closer to the levels of T than those of TD, except for 2-hexenal (*E*)-, indicating that the presence of α-tocopherol and d18:0 together was required for the lower volatile formation. According to the volatile oxidation product analysis, it does seem that some of the detected carbonyl compounds could have reacted with d18:0, resulting in significantly lower formation levels in TD when compared to T. However, the levels were lower in many cases also for the noncarbonyl compounds (e.g., alkenes and alcohols), suggesting that the better stability of the TD oil after 12 h oxidation (see Sections 3.1 and 3.2) could have affected the result. Additionally, the observed differences in the reaction routes of nonvolatile oxidation products most likely affected the differences. For example, the levels of direct LO^•^ cleavage products might be lower due to its reduction to more stable LOH.

### 3.5 Principal Component Analysis of Nonvolatile and Volatile Oxidation Products

**Figure 7:**
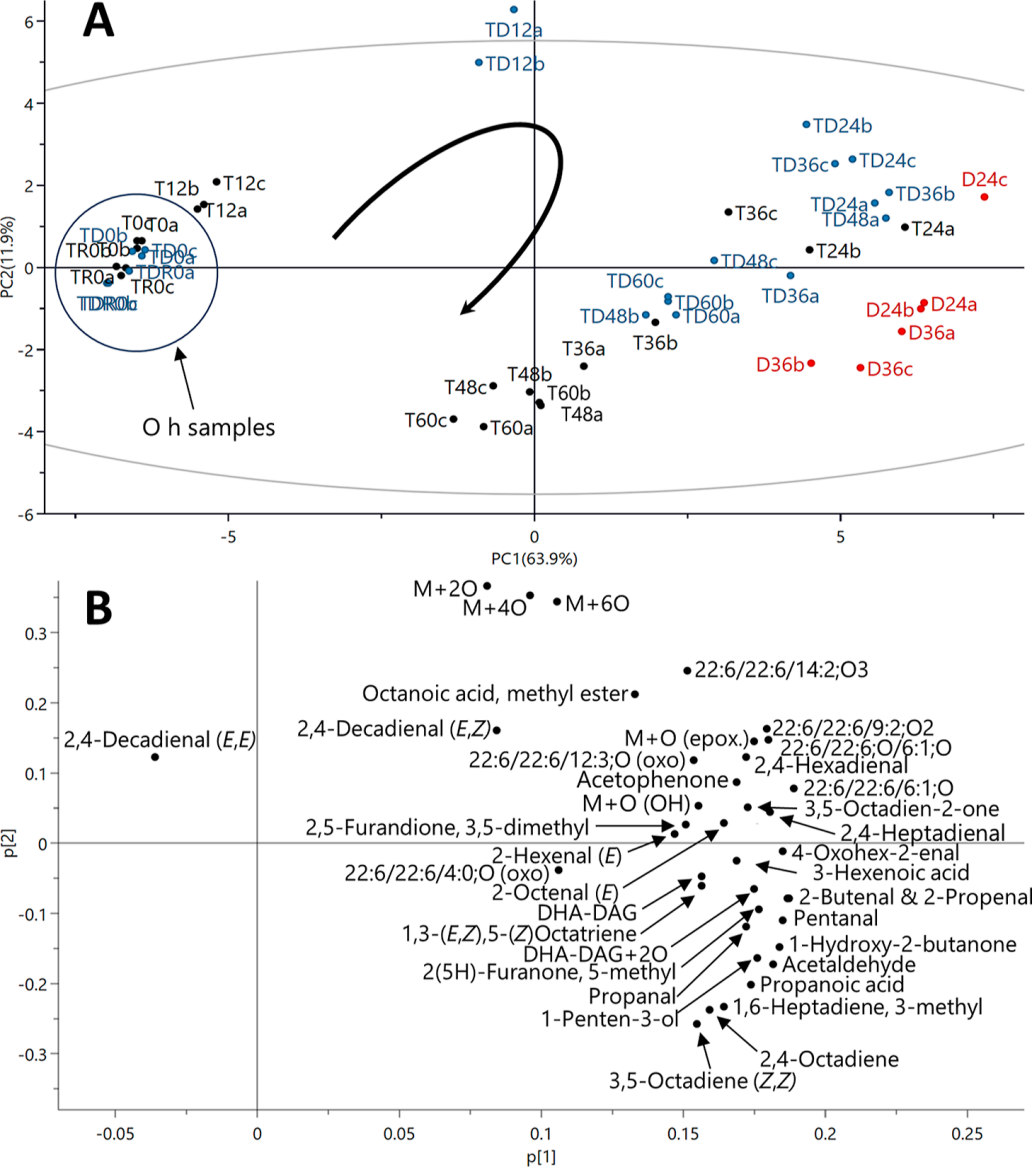
PCA model of UV scaled area data for the integrated volatile and nonvolatile oxidation products (PC1 vs PC2; R2X[1] = 0.639, Q2[1] = 0.617; R2X[2] = 0.119, Q2[2] = 0.204) with scores plot (A) and loadings plot (B) for DHA-TAG samples with α-tocopherol and d18:0 (TD, blue), α-tocopherol (T, black), and d18:0 (D, red) during the 60 h oxidation trial at 50 °C in the dark. The arrow in the scores plot (A) illustrates the direction of oxidation progress.

A PCA model was produced from the integrated area data to get an overall view of the oxidation progress and the grouping of oxidation products during the 60 h time frame (Figure 7). Both volatile and nonvolatile oxidation products were included, based on the areas of non-SPE-extracted samples. PC1 explained 63.9% and PC2 11.9% of the data variation. Only oxidation products present in all sample types were included in the model. The black arrow in the scores plot (Figure 7A) represents the direction of oxidation progress. The oxidation rates of the TD and T samples differentiated at 12 h, when the TD samples grouped together quite far at the positive side of PC2 and the T samples remained quite close to the nonoxidized zero samples at the negative side of PC1. As can be seen in the loadings plot (Figure 7B), the grouping of TD 12 h samples was mainly caused by the high levels of M + 2O, M + 4O, and M + 6O at 12 h. At 24 h, the T samples were already more oxidized (closer to the zero level of PC2) than TD samples, which was also seen earlier in the α-tocopherol and DHA depletion graphs (Figures 2A and 3, respectively). In the T samples, both 2,4-decadienals (*E*,*Z*/*E*,*E*)- were present in substantial amounts already at 0 h, and the decrease started at 12–24 h, which led to their grouping closer to the 0 and 12 h samples. Similarly, many of the nonvolatile acyl chain cleavage products peaked at 12–36 h and after that started to react more further, also causing their grouping on the positive side of PC2. Alkenes and propanoic acid had increasing levels until 48–60 h, and thus they grouped on the negative side of PC2 with the more oxidized samples. The D samples were grouping further from T and TD on the positive side of PC1. The maximum levels of DAG, DAG + 2O, alkenes, propanal, pentanal, and acetaldehyde were highest in the D samples, which probably affected their differentiation. Overall, the grouping of the oxidation products in the loadings plot roughly followed their formation time and longevity while the oxidation proceeded. The PCA results are in line with the other obtained data, showing faster oxidation for TD from 0 to 12 h and better preservation of DHA-TAG thereafter.

## 4 Discussion

The oxidation rate of TD samples was faster than that of T samples at the beginning of the oxidation trial from 0 to 12 h. This was shown in the nonvolatile and volatile oxidation product analysis as well as in the α-tocopherol and DHA levels. One possible cause for this might be the surface activity of d18:0, which led to droplet formation in TD oil, exposing a higher surface area against the air. In the T samples, the oil was mainly found as an unbroken surface at the bottom of the vial bottom. Previous studies on the synergistic antioxidative effect of d18:0 and α-tocopherol^10,24,25^ have used similar antioxidant concentrations but higher oil amounts (100–300 mg) and perhaps thus not experienced differences in droplet formation. After 12 h, when d18:0 was nearly consumed, the oxidation rate of TD samples slowed down, and the DHA-TAG with both d18:0 and α-tocopherol became better preserved than the oil with either compound alone. Improved stability was seen in the substantial reduction of DHA depletion rate (Figure 3), α-tocopherol consumption (Figure 2A), and lower levels of volatile oxidation products at 24 h (Figure 6). Contrary to volatile oxidation products, the levels of most nonvolatile oxidation products, especially the additions of oxygens to DHA-TAG, were higher in the TD samples than in T samples throughout the oxidation period (Figure 5). This indicated higher hydrogen availability in the TD samples, directing the reaction to an increased formation of hydroperoxides and hydroxides. Possible hydrogen sources in TD samples include the antioxidants formed in carbonyl–amine reactions and d18:0 amine groups.

In the case of D samples with just d18:0, the high volatile levels were not indicating consumption in carbonyl–amine reactions (Figure 6), although the LC-QTOF analysis of the SPE-extracted samples showed imine formation. DHA was also better preserved in D than in T samples at 24 and 36 h (Figure 3), and the d18:0 concentration had decreased below the LOD at 24 and 36 h (Figure 2B). In the D samples, d18:0 was the only direct H source in addition to the oil itself, so H transfer to lipid radicals was probably more common than in TD. Despite the high volatile levels, the results are indicating some antioxidant potential also for d18:0 alone. Contrary to the current study, Uemura et al.^24^ reported lower volatile levels in the d18:0-containing fish oil when compared to α-tocopherol and control samples. The antioxidant potential of d18:0 alone has not been observed in the oxygen concentration measurements of previous studies.^10,24^

The further reactions of d18:0 with the oxidation product carbonyls are tentatively shown. Also, a more stable condensation product of d18:0 and malondialdehyde (d18:0-MDA-d18:0) could be tentatively identified and semiquantified from the oxidized samples. These reactions also led to the formation of a yellow polymer in the more oxidized TD samples. The suspected antioxidative compounds, formed from further reactions of imines, could not be detected. Their unstable nature, low concentrations, and unknown structures challenge the detection and identification. The lower levels of several volatile oxidation products in the TD samples indicated participation in carbonyl–amine reactions. However, the better stability of TD oil and differing oxidation reaction routes of T, TD, and D probably also affected the results. There were indications that the presence of α-tocopherol was required for the formation and stabilization of some of the lipation products. Previous studies suggest that the presence of α-tocopherol is necessary for the antioxidative effect of d18:0 due to the mild oxidation conditions it provides.^10,24,25^ However, more research is needed to better understand the interactions behind the effect.

The obtained results support the hypothesis and agree with previous research, showing a synergistic antioxidative effect for d18:0 and α-tocopherol. To obtain a better understanding of the tentatively identified lipation reaction products, separate aldehyde-d18:0 reactions followed by structural and antioxidant efficacy analyses might be necessary. Inclusion of all time points also for d18:0 samples in subsequent studies would further clarify the antioxidant activity of d18:0 as such. In the current study, the used d18:0 concentration (1% w/w) was relatively high when considering current antioxidant applications for food/supplement use, and more research is needed on the effect of concentration on the antioxidant activity. Also, the effect of the temperature on the formation of antioxidative reaction products as well as their influence on sensory quality should be examined. Due to the amphiphilic property of d18:0, its antioxidant potential also in emulsions and other food systems could be a subject for future research. To conclude, this is the first study describing the formation of d18:0 imine structures in oxidizing oil. Also, the effect of d18:0 on the total oxidation pattern including numerous volatile and nonvolatile oxidation products is reported for the first time. At 1% (w/w) d18:0 and 0.05% (w/w) α-tocopherol concentration, the oxidation of DHA-TAG was strongly directed to increased formation/stabilization of hydroperoxides and hydroxides and lowered levels of volatile oxidation products.

## Supporting Information Available

Possible d18:0 imine structures detected in the UHLPC-QTOF analysis of the SPE-extracted samples; area evolution for the nonvolatile oxidation products M + 4O and M + 6O in DHA-TAG samples; and area evolution of the volatile oxidation products 2-propenal, 1-penten-3-ol, 1-hydroxy-2-butanone, 3-methyl-1,6-heptadiene, 3,5-octadien-2-one (*E*,*E*)-, and 3-hexenoic acid (*E*)- (PDF)

## Abbreviations

d18:0: dihydrosphingosine
DHA-TAG: tridocosahexaenoin
DHA: docosahexaenoic acid
TAG: triacylglycerol
L^•^: alkyl radical
LOO^•^: peroxyl radical
LOOH: lipid hydroperoxide
OH^•^: hydroxyradical
LO^•^: alkoxy radical
d18:1: D-*erythro*-sphingosine
LC: liquid chromatography
GC: gas chromatography
MS: mass spectrometry
SPE: solid-phase extraction
NP-UHPLC: normal-phase ultrahigh-performance liquid chromatography
FLD: fluorescence detection
FID: flame ionization detection
HS-SPME: headspace solid-phase microextraction
QTOF: quadrupole time-of-flight mass spectrometry
ESI: electrospray ionization
VSOP: volatile secondary oxidation product
PCA: principal component analysis
LOD: limit of detection
LOQ: limit of quantification
