# Supplementary material for: Antioxidative Effect of Dihydrosphingosine (d18:0) and α-Tocopherol on Tridocosahexaenoin (DHA-TAG)
Source: J Agric Food Chem. 2023 Sep 26;71(40):14769–81. doi: 10.1021/acs.jafc.3c02668 (PMC10571079; doi:10.1021/acs.jafc.3c02668)
Supplement: Supplementary file 1 — jf3c02668_si_001.pdf [file jf3c02668_si_001.pdf]

Antioxidative effect of dihydrosphingosine (d18:0) and α-tocopherol on tridocosahexaenoin (DHA-TAG)

Eija Ahonen*, Annelie Damerau, and Kaisa M. Linderborg*

Food Sciences, Department of Life Technologies, University of Turku, 20014 Turun yliopisto, Turku, Finland
*Correspondence: eija.s.ahonen@utu.fi (E. Ahonen) +358 29 450 5000; kaisa.linderborg@utu.fi (K.M. Linderborg) +358 50 439 5535.

Supporting information

Following supporting information is provided:

(1) Possible d18:0 imine structures detected in the UHLPC-QTOF analysis of the SPE extracted samples (Supplementary table 1.)
(2) Area evolution for the non-volatile oxidation products M+4O and M+6O in DHA-TAG samples (Supplementary figure 1.)
(3) Area evolution of the volatile oxidation products 2-propenal, 1-penten-3-ol, 1-hydroxy-2-butanone, 3-methyl-1,6-heptadiene, 3,5-octadien-2-one (*E*,*E*)-, and 3-hexenoic acid (*E*)- (Supplementary figure 2.)

Supplementary table 1. Possible d18:0 imine structures detected in the UHLPC-QTOF analysis of the SPE extracted samples. Atom arrangement in the carbonyl side can vary. Compound name, mass (*m/z*), adduct mass (*m/z*), retention time (min), and main fragments (*m/z*).

**Tentatively identified compound**	**Mass *m/z***	**Adduct *m/z***	**Adduct**	**RT min**	**Main fragments *m/z***
malondialdehyde-d18:0	355.311	356.319	$[M+H]^+$	6.60	338.308_320.297_292.303_72.048_114.059_254.288
glyoxal-d18:0	341.296	342.304	$[M+H]^+$	8.00	296.296_284.297_254.288_266.285
oxobutenal-d18:0	367.310	368.318	$[M+H]^+$	8.12	350.307_ 284.297_322.312_266.285_84.048
oxohexadienal-d18:0	393.327	394.335	$[M+H]^+$	8.40	378.323_338.308_346.313_110.063_153.081_266.286
propanal-d18:0	341.331	342.338	$[M+H]^+$	8.50	284.297_313.320_212.168_266.285
oxo/epoxypentenal-d18:0	381.328	382.335	$[M+H]^+$	8.51	364.324_338.308_266.28_284.297
2-propenal-d18:0	339.316	340.324	$[M+H]^+$	9.48	284.297_266.285_322.314_313.320_212.167_129.094
2,4-decadienal-d18:0	435.410	436.418	$[M+H]^+$	11.00	266.285_284.297_388.398

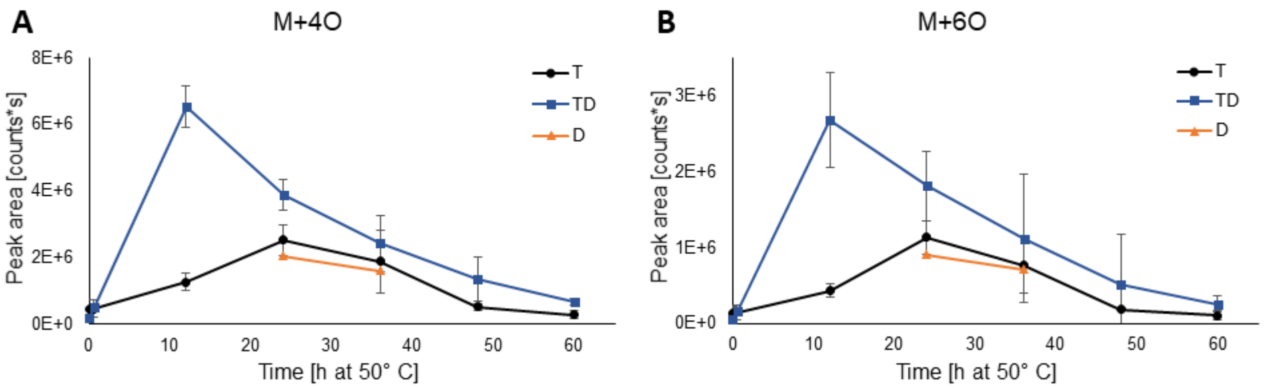

Supplementary figure 1. Area evolution for the non-volatile oxidation products M+4O (A) and M+6O (B) in DHA-TAG samples with α-tocopherol and d18:0 (TD, blue line, square marker), α-tocopherol (T, black line, round marker), and d18:0 (D, orange line, triangle marker) during the 60-h oxidation trial at 50° C in the dark.

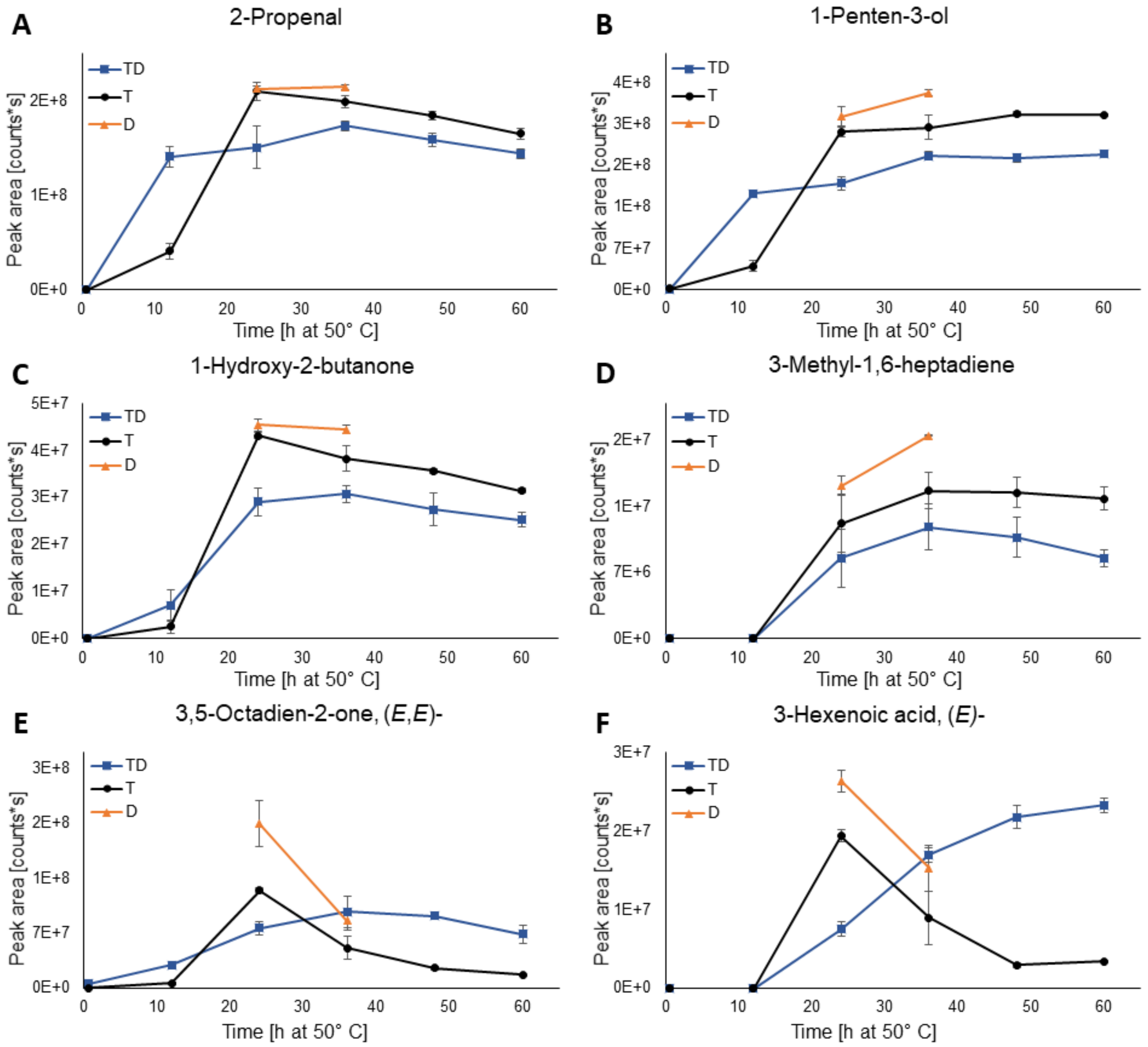

Supplementary figure 2. Area evolution of the volatile oxidation products 2-propenal (A), 1-penten-3-ol (B), 1-hydroxy-2-butanone (C), 3-methyl-1,6-heptadiene (D), 3,5-octadien-2-one, (*E*,*E*)- (E), and 3-hexenoic acid, (*E*)- (F) for the DHA-TAG samples with α-tocopherol and d18:0 (TD, blue line, square marker), α-tocopherol (T, black line, round marker), and d18:0 (D, orange line, triangle marker) during the 60-h oxidation trial at 50° C in the dark.
